# The Confusion in Renaming Species: Penicillium chrysogenum and Penicillium rubens

**DOI:** 10.1128/MRA.00464-21

**Published:** 2021-12-02

**Authors:** Ulrich Kück, Tim A. Dahlmann

**Affiliations:** a Allgemeine und Molekulare Botanik, Ruhr-Universität, Bochum, Germany; Vanderbilt University

## REPLY

Houbraken et al. (1) mention our genome sequencing work on strain P2niaD18 and claim that we have incorrectly identified this strain. We previously published data (2, 3) on strain Q176, the ancestor strain of P2niaD18, and referred to the 2011 paper by Houbraken et al. (4), which discussed the renaming of Penicillium chrysogenum to Penicillium rubens. However, the Seventeenth International Botanical Congress (Vienna, Austria; July 2005) (5) suggested that traditional taxonomic identifications should be retained for simplification and clarity. Indeed, *P. rubens*, the renamed P. chrysogenum, has often appeared in recent publications by diverse groups, but all of them used the traditional name P. chrysogenum (6–9). We expect that species name changes attached to taxonomic names will get updated over time, thus reflecting the ever-changing nature of taxonomic nomenclature.

We ourselves have shown very recently that based on beta-tubulin sequences, P2niaD18 and Pc3 are *P. rubens* species (10). But when we used the sequence of the sex-determining mating type loci, *P. rubens* and P. chrysogenum strains were not distinguishable, thus suggesting that both species are only distinguishable by minor morphological and/or molecular differences.

In summary, we appreciate the careful survey conducted by Houbraken and coworkers, all of whom are excellent taxonomists of moldy ascomycetes. However, we would like to ask them to amend their statement of “incorrect identification,” with one that simply mentions that the species were “renamed.”

We foresee that in the international literature, penicillin-producing strains will continue to be called Penicillium chrysogenum, since all industrial strains can be traced back to a common ancestor, the wild-type strain NRRL 1951 (CBS 307.48), as was outlined in previous papers (8, 11).

We do not want to start a major dispute about the taxonomy of penicillin production strains such as *P. rubens* but would rather like to close with a Shakespearean quote from the tragedy *Romeo and Juliet*: “That which we call a rose, by any other name would smell as sweet.”

## ACKNOWLEDGMENT

Funded by the German Research Foundation (DFG), project KU 517/15-1.
